# Correction: A new topology of the HK97-like fold revealed in Bordetella bacteriophage by cryoEM at 3.5 Å resolution

**DOI:** 10.7554/eLife.07482

**Published:** 2015-03-18

**Authors:** Xing Zhang, Huatao Guo, Lei Jin, Elizabeth Czornyj, Asher Hodes, Wong H Hui, Angela W Nieh, Jeff F Miller, Z Hong Zhou

Zhang X, Guo H, Jin L, Czornyj E, Hodes A, Hui WH, Nieh AW, Miller JF, Zhou ZH. 2013. A new topology of the HK97-like fold revealed in Bordetella bacteriophage by cryoEM at 3.5 Å resolution. *eLife* **2**:e01299. doi: 10.7554/eLife.01299. Published 17 December 2013

The authors declare the following competing interest, which should have been declared at the time of publication.

Jeff F Miller is a cofounder, equity holder, and chair of the scientific advisory board of AvidBiotics Inc., a biotherapeutics company based in San Francisco. The other authors declare that no competing interests exist.

The article has been corrected accordingly.

